# Porphyria Cutanea Tarda: A Multifactorial Disease

**DOI:** 10.7759/cureus.87965

**Published:** 2025-07-15

**Authors:** Ana Maria Baltazar, Liliia Savka, Nuno R Carreira

**Affiliations:** 1 Medical Department, Hospital de Santa Maria, Unidade Local de Saúde de Santa Maria, Lisboa, PRT

**Keywords:** hfe mutation, iron overload, porphyria cutanea tarda, porphyrin metabolism, primary sjögren syndrome

## Abstract

Porphyria cutanea tarda (PCT) is a disorder characterized by porphyrin accumulation leading to photosensitivity and skin fragility, often triggered by a combination of genetic, autoimmune, and environmental factors. This case describes a 39-year-old woman with primary Sjögren syndrome (pSS) and heterozygosity for the HFE H63D mutation who developed PCT following hydroxychloroquine (HCQ) therapy. Initial symptoms included xerostomia, fatigue, and skin lesions. Laboratory tests showed liver enzyme elevation, leukopenia, hyperferritinemia, and positive anti-SSA/SSB antibodies. Hydroxychloroquine-induced hepatotoxicity prompted treatment discontinuation, and porphyrin analysis confirmed PCT. Despite the absence of UROD gene mutations, the interplay between pSS, iron overload, and medication exposure supported a diagnosis of sporadic PCT. The patient was successfully managed with low-dose HCQ for porphyrin clearance and azathioprine for autoimmune disease control. This case highlights the importance of recognizing multifactorial contributors in PCT and tailoring treatment accordingly to avoid complications.

## Introduction

Porphyrias are a group of rare metabolic disorders resulting from enzymatic deficiencies in the heme biosynthesis pathway, leading to the accumulation of toxic precursors such as aminolevulinic acid (ALA) and porphobilinogen (PBG). These conditions can be classified into genetic (hereditary) and acquired porphyrias, each with distinct characteristics [1].

Hereditary porphyrias are caused by mutations in genes encoding specific enzymes involved in heme synthesis. These mutations can be transmitted from parents to offspring, following autosomal dominant, autosomal recessive, or X-linked inheritance patterns. Among the hereditary porphyrias, the following are noteworthy [2]: acute intermittent porphyria (AIP) is marked by neurovisceral crises, such as severe abdominal pain and neurological symptoms, and is inherited in an autosomal dominant manner; hereditary coproporphyria (HCP) presents with symptoms similar to AIP, including abdominal pain and neurological issues, and follows an autosomal dominant inheritance pattern; variegate porphyria (VP) features a combination of neurovisceral and cutaneous symptoms and is likewise inherited in an autosomal dominant fashion; congenital erythropoietic porphyria (CEP) is distinguished by severe skin-related symptoms from birth and follows an autosomal recessive pattern of inheritance.

The primary form of acquired porphyria is porphyria cutanea tarda (PCT). Although there is a hereditary form (PCT type 2), PCT may result from environmental factors that inhibit the activity of the enzyme uroporphyrinogen decarboxylase (UROD), leading to porphyrin accumulation (PCT type 1). Triggering factors include excessive alcohol consumption - alcohol can precipitate porphyria symptoms, particularly in predisposed individuals; estrogen exposure - hormonal therapies containing estrogens may trigger symptoms in susceptible individuals; viral infections - hepatitis C is the viral infection most commonly associated with the development of PCT, though HIV may also play a role; iron overload (including hemochromatosis) - excess iron in the body can inhibit UROD activity, contributing to PCT development; autoimmune diseases such as systemic lupus erythematosus [3].

PCT is the most common subtype of porphyria [4], characterized by the accumulation of porphyrins mainly in the skin, which leads to photosensitivity, blistering, and hyperpigmentation, especially in sun-exposed areas. PCT is commonly associated with hepatic dysfunction, due to porphyrin-mediated hepatocyte injury. Iron overload may be seen in patients with mutations in the HFE gene, which is involved in iron metabolism. Studies have shown that the HFE H63D and C282Y mutations predispose individuals to iron accumulation, further increasing the risk of porphyric episodes [5]. Additionally, PCT may develop as a multifactorial disorder triggered by environmental factors, infections, and certain medications such as oral contraceptives and antimalarials.

Although the association between PCT and autoimmune diseases is rare, there are reports in the literature documenting the coexistence of PCT with systemic lupus erythematosus, and more rarely, the coexistence of PCT and Sjögren's syndrome. It is important to note that the concomitant presence of PCT and autoimmune diseases can complicate clinical management, especially regarding the use of medications that may trigger porphyria crises. Therefore, it is essential for healthcare professionals to be aware of these potential associations to ensure accurate diagnosis and appropriate treatment.

Hydroxychloroquine (HCQ), a common treatment for connective tissue diseases, is known to facilitate skin and other tissue porphyrin clearance in low doses but may exacerbate liver toxicity when administered at full doses in individuals with porphyric predispositions [6].

The coexistence of primary Sjögren's syndrome (pSS), an autoimmune condition linked to chronic inflammation and mild liver dysfunction, adds complexity to PCT management.

## Case presentation

We present the case of a 39-year-old woman referred to an internal medicine appointment due to complaints of dry mouth (xerostomia), recurrent oral aphthous ulcers, dry eyes (xerophthalmia), malaise, and fatigue. She worked as an insurance agent, and her medical history was significant only for migraines. She had no history of surgeries, smoking habits, or recreational drugs. Her alcohol consumption was minimal, and she denied using supplements or herbal products. Living in an urban area, she had not traveled internationally, and her vaccinations were up to date, including hepatitis B.

Her medications included a combined oral contraceptive pill (drospirenone + ethinylestradiol) and an acetaminophen-caffeine combination for migraines. Her symptoms of xerostomia, xerophthalmia, malaise, and fatigue had begun eight months prior, leading to blood tests revealing leukopenia (2.67 x 10^9/L), neutropenia (1.00 x 10^9/L), and mildly elevated liver enzymes: aspartate aminotransferase (AST; 83 U/L), alanine aminotransferase (ALT; 125 U/L), and γ-glutamyltransferase (γGT; 46 U/L). HIV and hepatitis C screening were negative, and she had antibodies against hepatitis B as well as a serology consistent with a prior hepatitis A infection. She showed elevated ferritin (620 ng/mL), an inflammatory protein electrophoresis pattern, and normal ceruloplasmin and thyroid function.

Abdominal ultrasound revealed a normal-sized liver with heterogeneous echotexture and steatosis. Suspecting hemochromatosis, genetic testing was conducted, showing heterozygosity for the H63D mutation in the HFE gene.

Her symptoms progressively worsened, limiting her ability to work or perform physical activities, prompting a referral to an internal medicine appointment. In addition to her previous symptoms, she reported weight loss (10% of body weight in a month, BMI 17.9 kg/m²), alopecia, facial hypertrichosis, hyperpigmented skin, skin fragility with painful blistering, and scarring, particularly on sun-exposed areas like the hands and arms. She occasionally experienced a dry cough but denied other symptoms such as photosensitive rash, erythema, urticaria, or arthritis. She had no history of current or prior pregnancy, including miscarriage.

**Figure 1:**
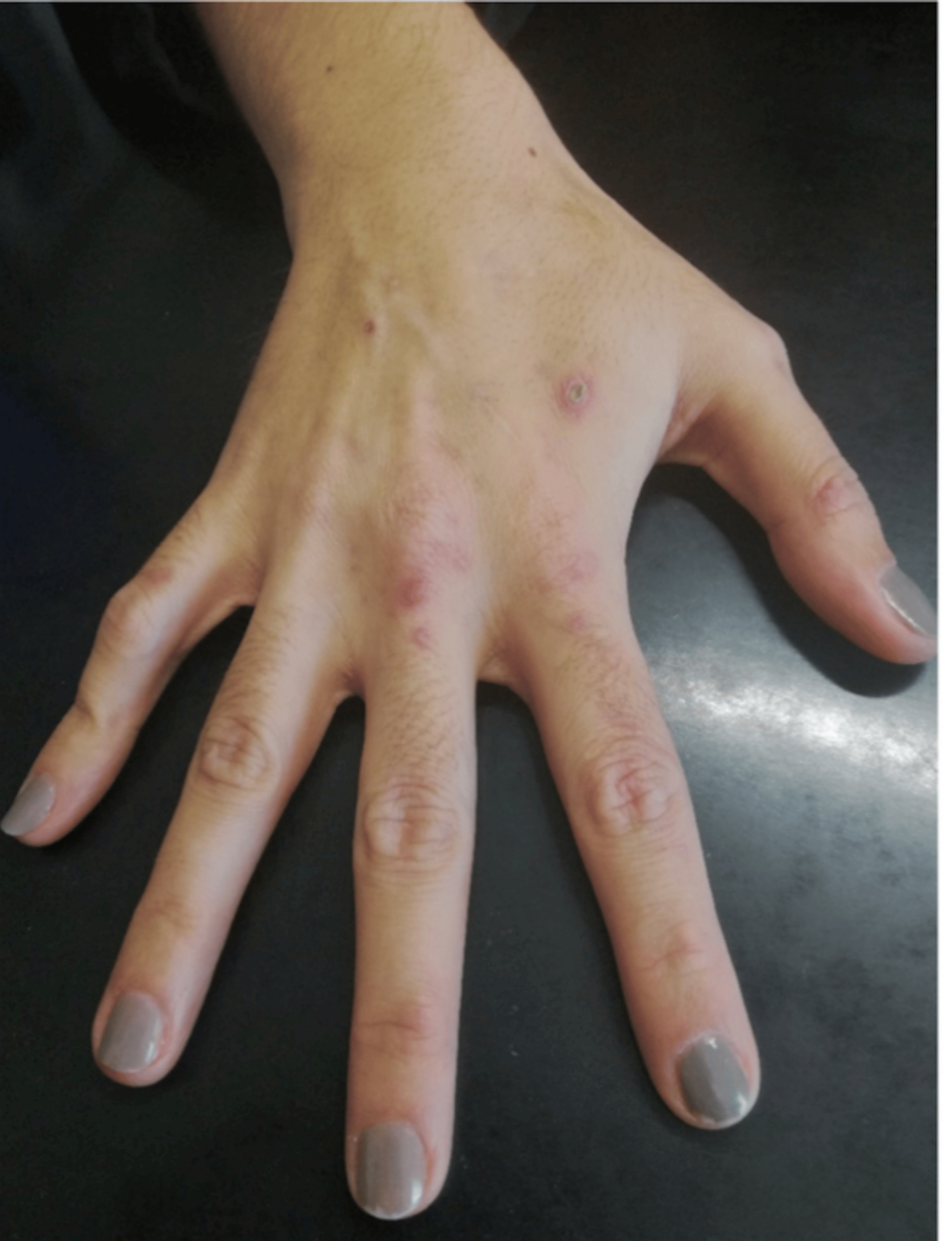
Dermatologic findings Multiple skin lesions - vesicular, ulcerated, and scarred - in different stages of progression (photograph taken with the patient’s permission).

Physical examination revealed a slightly underweight, normotensive, afebrile patient with a normal heart rate and oxygen saturation. Cardiopulmonary examination was unremarkable. On physical assessment, marked facial hypertrichosis and skin lesions were evident (Figure 1).

A comprehensive blood workup was conducted, including autoantibody panel, complement levels, tuberculosis screening, and other infectious serologies. In addition to leukopenia and neutropenia, she had mild anemia (hemoglobin 11.8 g/dL), elevated ferritin, and mildly increased AST, ALT, and γGT. Autoimmune workup revealed positive antinuclear antibodies (ANA 1:320, fine speckled), positive anti-SSA/Ro52, and notably positive anti-SSB/Ro60 antibodies, while other autoantibodies were negative. Alpha-1 antitrypsin was within normal limits. Thoracic imaging and electrocardiogram were unremarkable, as were cardiac and pulmonary evaluations. Labial salivary gland biopsy indicated focal sialadenitis, confirming a diagnosis of pSS per the 2016 American College of Rheumatology (ACR)-European Alliance of Associations for Rheumatology (EULAR) criteria.

One month later, she began treatment with hydroxychloroquine (400 mg daily) and prednisolone (5 mg daily). However, on the third day of therapy, she developed fever with chills, nausea, vomiting, and dark urine. Physical examination revealed stable vital signs except for a fever of 38.5 ºC. Abdominal examination remained unremarkable.

Follow-up blood tests showed further elevation of AST, ALT, and lactate dehydrogenase, alongside a slight increase in C-reactive protein, without signs of hepatic insufficiency. Hydroxychloroquine and prednisolone were discontinued, and subsequent blood work indicated a temporary worsening of liver enzymes, which later returned to baseline (Table 1).

**Table 1:** Laboratory findings Sequential lab results showing hepatic enzymes elevation after introducing hydroxychloroquine on 07/07/2018. AST – Aspartate Aminotransferase, ALT – Alanine Transaminase, ALP – Alkaline Phosphatase, GGT – γ-Glutamyltransferase, LDH – Lactate Dehydrogenase, INR – International Normalized Ratio Test

	Reference range	14/june	10/july	11/july	13/july	17/july
White Blood Cell Count	(4.5-5.9 x 10^9/L)	2.21x10^9^/L	4.52x10^9^/L	3.11x10^9^/L	3.44x10^9^/L	2.89x10^9^/L
Platelets	(150-450 x 10^9/L)	198x10^9^/L	164x10^9^/L	146x10^9^/L	162x10^9^/L	236x10^9^/L
C-Reactive Protein	(<0.5 mg/dL)	0.130mg/dL	2.91 mg/dL	5.36 mg/dL	1.47 mg/dL	1.47 mg/dL
AST	(0-40 U/L)	68 U/L	576 U/L	744 U/L	205 U/L	92 U/L
ALT	(0-41 U/L)	102 U/L	559 U/L	877 U/L	515 U/L	210 U/L
ALP	(40-130 U/L)	69 U/L	75 U/L	99 U/L	80 U/L	-
GGT	(0-60 U/L)	55 U/L	53 U/L	49 U/L	78 U/L	81 U/L
Total Bilirubin	(<1.2 mg/dL)	0.47 mg/dl	0.90 mg/dL	0.74 mg/dL	0.51 mg/dL	0.41 mg/dL
LDH	(100-250 U/L)	178 U/L	513 U/L	585 U/L	242 U/L	220 U/L
INR	-	0.95	1.19	-	0.99	0.92

Further evaluation excluded acute viral hepatitis, and repeat abdominal ultrasound showed persistent steatosis. Two weeks later, a liver biopsy revealed acute hepatitis with lymphoplasmacytic infiltrates, chronic steatohepatitis, and mild to moderate hemosiderosis, but no features of autoimmune liver disease such as primary biliary cholangitis.

The constellation of skin findings, hypertrichosis, hyperpigmentation, and liver injury, likely hydroxychloroquine-induced, a patient with HFE H63D heterozygosity and pSS raised suspicion for PCT. Plasmatic, urinary, and fecal porphyrins showed elevated total porphyrins, uroporphyrin I and III, pentacarboxyporphyrin, and coproporphyrin I, confirming the diagnosis (Table 2).

**Table 2:** Plasmatic, urinary, and fecal porphyrins: patient's measurements

Porphyrins	Plasmatic	Reference range	Urinary	Reference range	Fecal	Reference range
Total Porphyrins	14.5 ug/L	<32.5 ug/L	324 ug/24h	<150 ug/24h	48.1 ug/g	<23.8 ug/g
Uroporphyrin (Uroporphyrin I and III)	8.4 ug/L	<11.8 ug/L	241 ug/24h	<25 ug/24h	8.8 ug/g	<5 ug/g
Hepta-Carboxylic Porphyrin	0.3 ug/L	<3.8 ug/L	5 ug/24h	<5 ug/24h	0.9 ug/g	<0.8 ug/g
Hexa-Carboxylic Porphyrin	<0.1 ug/L	<1.3 ug/L	2 ug/24h	<5 ug/24h	0.4 ug/g	<0.7 ug/g
Penta-CarboxylicPorphyrin	4.0 ug/L	<1.2ug/L ug/L	29 ug/24h	<5 ug/24h	18.2 ug/g	<0.6 ug/g
Coproporphyrin I	1.0 ug/L	<6.4ug/L ug/L	41 ug/24h	<25 ug/24h	15.3 ug/g	<8.7 ug/g
Coproporphyrin III	0.8 ug/L	<8.0 ug/L	6 ug/24h	<75 ug/24h	4.5 ug/g	<8.0 ug/g

Next-generation sequencing for nine porphyria-associated genes (ALAD, ALAS2, CPOX, FECH, GATA1, HMBS, PPOX, UROD, UROS) revealed no mutations, particularly in the UROD gene. Despite the absence of UROD mutations, hyperferritinemia, likely due to HFE heterozygosity and pSS, contributed to the final diagnosis of sporadic (type 1) PCT.

Hydroxychloroquine 200 mg twice weekly is effective for PCT by reducing skin porphyrin levels, but at higher doses, it is porphyrinogenic and potentially hepatotoxic due to the overload of porphyrins in the liver. The patient’s PCT was effectively managed with low-dose hydroxychloroquine for its porphyrin-lowering effects. A multidisciplinary team, including dermatology and hepatology, advised against all porphyrinogenic medications due to her prior liver injury. Her pSS was managed with azathioprine (1.5 mg/kg) and prednisolone (5 mg), leading to symptom remission and normalization of blood cell counts. For skin lesions, the patient was advised to apply broad-spectrum sunscreen, zinc oxide, and retinoids. Due to a worsening of the cutaneous lesions, hydroxychloroquine 200 mg twice weekly was initiated, leading to lesion remission. 

## Discussion

The case presented here is a unique example of a multifactorial disorder involving both genetic and autoimmune components. The patient, who carries the HFE H63D mutation, has a predisposition to iron overload, which, combined with the autoimmune condition, pSS, led to the development of PCT [3]. The patient’s clinical presentation, which included skin fragility, hyperpigmentation, and photosensitivity, alongside liver dysfunction and elevated ferritin levels, raised suspicion for PCT. This complex interplay between genetic mutations, autoimmune disease, and environmental factors highlights the importance of a multidisciplinary approach in diagnosing and managing such cases.

**Figure 2:**
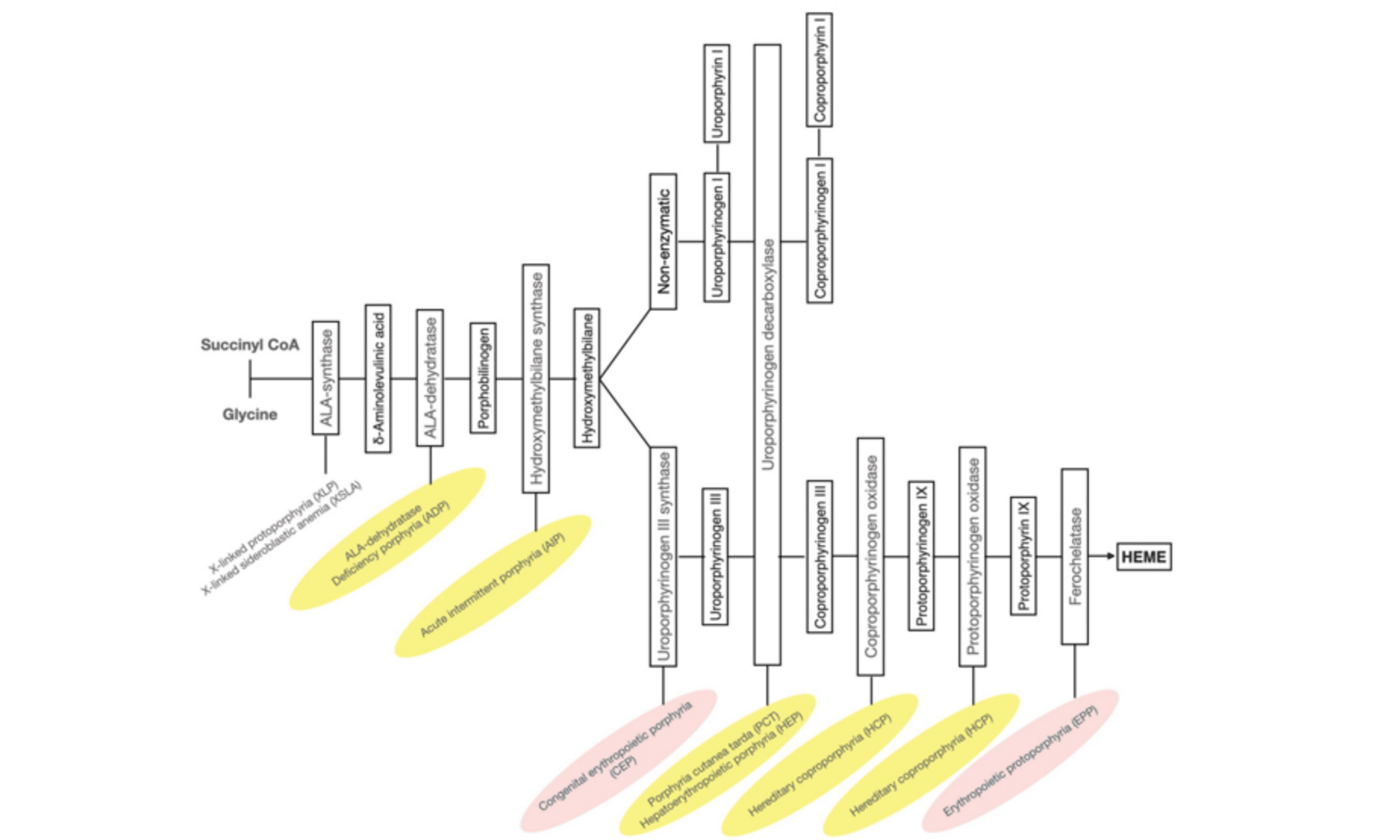
Heme biosynthetic pathway The human heme biosynthetic pathway indicates in linked boxes the enzyme that, when deficient, causes the respective porphyria. Hepatic porphyrias are shown in yellow boxes and erythropoietic porphyrias in pink boxes. Adapted from [8]

The coexistence of PCT with autoimmune diseases is rare but documented in the literature, with systemic lupus erythematosus and Sjögren’s syndrome being the most frequently associated conditions [7]. In this case, the patient’s positive anti-SSA/Ro52 and anti-SSB/Ro60 antibodies, combined with a minor salivary gland biopsy, confirmed the diagnosis of pSS, further complicating her clinical management. Autoimmune diseases like pSS can lead to chronic inflammation and mild liver dysfunction, which, when combined with genetic mutations like HFE H63D, increases the risk of developing PCT. The fact that the patient did not show mutations in the UROD gene, despite the clinical signs of PCT, suggests that her PCT was triggered by environmental and other genetic factors, rather than being a purely hereditary form (Figure 2) [8]. This emphasizes the multifactorial nature of PCT, where genetic predispositions, such as HFE mutations, interact with autoimmune conditions and environmental triggers to manifest the disease.

The role of medications, particularly HCQ, in triggering or exacerbating PCT must also be considered in clinical management. HCQ, commonly used to treat autoimmune diseases like SLE and pSS, has been reported to facilitate porphyrin clearance at low doses. However, at higher doses, it can exacerbate liver toxicity and trigger porphyria crises in predisposed individuals. In this case, the patient experienced a worsening of liver function following the initiation of HCQ, which was promptly discontinued. This adverse reaction is consistent with previous reports that suggest caution when prescribing HCQ to patients with PCT or a porphyric predisposition.

**Figure 3:**
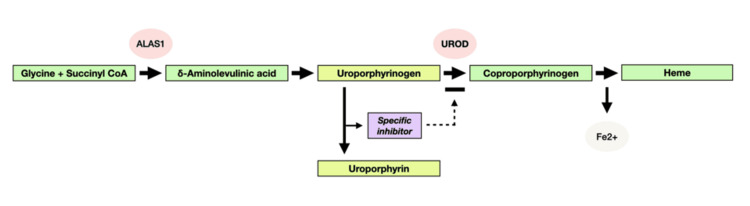
Generation of a hepatic UROD inhibitor in PCT Schema for the generation of a specific inhibitor of uroporphyrinogen decarboxylase in the liver in the presence of iron, cytochrome P450 enzymes (especially CYP1A2), and oxidative stress in PCT Adapted from [9] PCT: porphyria cutanea tarda; UROD: uroporphyrinogen decarboxylase

**Figure 4:**
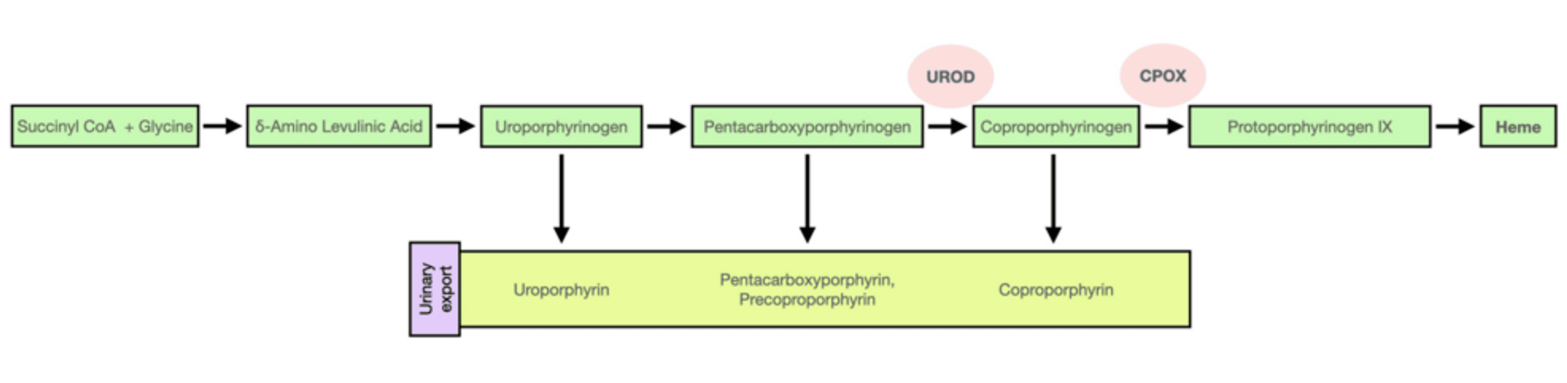
Pathway of heme synthesis Pathway of heme synthesis, major urinary metabolites, and inhibition by heavy metals Adapted from [10]

Additionally, the patient’s elevated ferritin levels and mild liver dysfunction were indicative of iron overload, a common finding in individuals with HFE mutations. Iron overload contributes to the development of PCT by inhibiting the activity of the enzyme UROD (Figure 3), thus promoting the accumulation of porphyrins in the skin [9]. The patient’s H63D mutation in the HFE gene may have played a crucial role in this process, further emphasizing the importance of recognizing genetic factors in the pathogenesis of acquired forms of PCT (Figure 4) [10].

Phlebotomy, a standard therapeutic approach in PCT to reduce hepatic iron overload and restore UROD activity, was not initiated in this case due to relative contraindications. The patient presented with mild normocytic anemia (Hb 11.8 g/dL), a low body mass index (BMI 17.9 kg/m²), and serum ferritin levels below the commonly accepted threshold for initiating phlebotomy (>1000 ng/mL). In such scenarios, low-dose, intermittent hydroxychloroquine serves as an effective alternative, promoting hepatic porphyrin mobilization and excretion with a lower risk of hepatotoxicity or hematologic adverse effects.

The management of this patient required a careful balancing of treatments for both her autoimmune disease and PCT. Hydroxychloroquine was substituted with azathioprine, a more suitable medication for pSS in this context. Furthermore, the avoidance of porphyrinogenic medications was recommended, and alcohol withdrawal was advised, given the patient’s history of liver injury.

This case illustrates the complex nature of PCT, which can arise from a combination of genetic mutations, autoimmune diseases, chronic infections, and environmental triggers. It also underscores the importance of early diagnosis and a tailored approach to treatment, especially when managing a multifactorial disorder like PCT. Healthcare professionals must be vigilant in identifying potential triggers, such as medications and iron overload, and should consider the genetic background of patients to ensure appropriate management and minimize complications.

## Conclusions

This case underscores the need for careful management of patients with multifactorial conditions like PCT, especially those with genetic mutations and concurrent autoimmune diseases. The presence of an HFE mutation, pSS, and the hepatotoxic reaction to hydroxychloroquine highlights the complexity of treatment planning in such cases. Although low-dose hydroxychloroquine is generally effective for PCT management, this case demonstrates the risks of standard-dose therapy in patients with underlying hepatic vulnerability. Azathioprine and low-dose corticosteroids were successfully implemented for managing the pSS without further hepatic compromise, illustrating the importance of personalized treatment plans.

Given this patient's unique presentation, including HFE mutation, pSS, and PCT, this case provides insights into managing a multifactorial disorder involving genetic, autoimmune, and medication-related factors. Further research is needed to clarify the mechanisms of drug-induced porphyria exacerbations in genetically susceptible individuals, which may lead to better guidelines for managing similar cases.
